# Mitochondria associated membranes in dilated cardiomyopathy: connecting pathogenesis and cellular dysfunction

**DOI:** 10.3389/fcvm.2025.1571998

**Published:** 2025-03-17

**Authors:** Pingge He, Hongbo Chang, Yueqing Qiu, Zhentao Wang

**Affiliations:** ^1^ Second School of Clinical Medicine, Henan University of Chinese Medicine, Zhengzhou, China; ^2^ Department of Cardiovascular Medicine, Second Affiliated Hospital of Henan University of Chinese Medicine, Zhengzhou, China

**Keywords:** MAMS, DCM, MAMs-associated proteins, cardiomyocytes, mitochondrial dysfunction

## Abstract

Dilated cardiomyopathy (DCM) is a leading cause of heart failure, yet therapeutic options remain limited. While traditional research has focused on mechanisms such as energy deficits and calcium dysregulation, increasing evidence suggests that mitochondria-associated membranes (MAMs) could provide new insights into understanding and treating DCM. In this narrative review, we summarize the key role of MAMs, crucial endoplasmic reticulum (ER)-mitochondria interfaces, in regulating cellular processes such as calcium homeostasis, lipid metabolism, and mitochondrial dynamics. Disruption of MAMs function may initiate pathological cascades, including ER stress, inflammation, and cell death. These disruptions in MAM function lead to further destabilization of cellular homeostasis. Identifying MAMs as key modulators of cardiac health may provide novel insights for early diagnosis and targeted therapies in DCM.

## 1 Introduction

Dilated cardiomyopathy (DCM) is a progressive disease characterized by left ventricular enlargement and impaired contractile function, often caused by genetic mutations, viral infections, and autoimmune diseases (1). The pathophysiology of DCM includes myocardial fibrosis, cardiomyocyte hypertrophy, apoptosis, and contractile dysfunction, influenced by factors including genetic mutations [e.g., Troponin T2, Cardiac Type, Titin, Lamin A/C(LMNA), Phospholamban (PLN), Tropomyosin 1, Laminin Subunit Alpha 2], myocardial injury, ventricular remodeling, oxidative stress, and mitochondrial dysfunction, which ultimately leads to heart failure (2). Current treatments, including pharmacological therapies (e.g., angiotensin-converting enzyme inhibitors, β-blockers, andSodium-Glucose Cotransporter 2 inhibitors) and device interventions (e.g., implantable cardioverter-defibrillators, left ventricular assist devices), remain limited by their inability to reverse cardiac damage or halt disease progression, underscoring the need for novel therapeutic strategies (3).

Mitochondria and the endoplasmic reticulum (ER) are central to DCM pathogenesis, regulating energy metabolism, calcium homeostasis, and apoptosis. Mitochondria produce ATP essential for cardiac contraction, while the ER ensures calcium storage and protein folding (4, 5). In DCM, dysfunction in these organelles leads to energy deficits, calcium dysregulation, and cellular stress responses, exacerbating cardiac dysfunction (6).

According to recent studies, mitochondria-associated membranes (MAMs), physical connections of the ER and mitochondria, serve as a platform for signal transduction and inter-organelle communication (7). MAMs facilitate calcium transfer, regulate mitochondrial ATP production, and prevent calcium overload-induced apoptosis (8, 9). They are also implicated in ER stress, mitochondrial dynamics, autophagy, inflammation, and oxidative stress, all of which contribute to DCM (10–12). Despite their importance, the precise mechanisms by which MAMs influence DCM remain poorly understood, and their therapeutic potential is largely unexplored. This review aims to elucidate the structure and function of MAMs, summarize their role in DCM development, and highlight their potential as a novel therapeutic target for this complex disease.

## 2 Structural characteristics and functional implications of MAMs

MAMs were first reported in the late 1950s and were successfully isolated from rat liver tissue in the 1990s (13). Advances in techniques such as transmission electron microscopy have confirmed that MAMs are subcellular compartments located at the mitochondria–ER contact (MERC) sites, consisting of protein complexes within the gap between the ER and the outer mitochondrial membrane (OMM). These complexes physically link the membranes of the two organelles while maintaining a defined distance, providing a structural platform for inter-organellar communication. The number, width, and length of MAMs are regulated parameters that reflect various physiological and pathological mechanisms (14). For instance, the number of MAMs increases by 2.5-fold during early ER stress in Henrietta Lacks cell (15), and their abundance is also elevated under hypoxic conditions (16). Typically, the distance between the OMM and smooth ER is maintained at 10–25 nm, while the distance from ribosome-rich rough ER is 50–80 nm (17). This distance is dynamic and varies with cellular metabolic states; narrower gaps enhance ER-mitochondria interactions, while wider gaps weaken them (18). For example, lipid transfer often occurs within ultra-tight contacts of <10 nm (19), and apoptotic stimuli reduce the MERC distance in Rat Basophilic Leukemia 2H3 cells from 28.2 to 20 nm (18). MAMs cover varying proportions of the mitochondrial surface depending on cell type, typically ranging from 4% to 20%, and within the same cell type, their coverage varies with stress and metabolic states (20). When the liver is in the postprandial state, the average length of MERC nearly doubles, with the coverage increasing from 4% to 11%, facilitating the metabolic adaptations required for postprandial liver function (20).

To date, in-depth mass spectrometry investigations have revealed over 1,000 proteins associated with MAMs in the brain and liver (21). MAM-associated proteins are categorized into three types based on their cellular localization: (1) proteins that are only found on the MAMs; (2) proteins that are expressed in both the MAMs and other cellular compartments; and (3) proteins that accumulate on the MAMs under specific conditions (22). Resident proteins within MAMs are characterized according to their particular activities. Calcium-regulatory proteins facilitate calcium ion flow between the ER and mitochondria, which is required for mitochondrial ATP generation and cellular stress adaptation (4). Lipid transport proteins aid in the transfer of phospholipids, such as phosphatidylserine and phosphatidylethanolamine, hence promoting mitochondrial membrane integrity and bioenergetic functions (19). Structural anchoring proteins keep mitochondria and the ER in close proximity, facilitating inter-organellar communication and coordinating activities including mitochondrial fusion, fission, and autophagy (11). In addition to their structural functions, MAMs are important regulators of inflammation, oxidative stress, and cell death (23). Dysregulation of calcium homeostasis at MAMs can lead to mitochondrial calcium excess, apoptosis, and energy deficits (4). Similarly, decreased lipid transport can undermine mitochondrial membrane stability, resulting in cardiomyocyte dysfunction (24). These disturbances are directly linked to the pathogenesis of many cardiovascular illnesses, including ventricular hypertrophy, diabetic cardiomyopathy, and myocardial infarction (11). For example, increased calcium signaling at MAMs exacerbates ventricular hypertrophy (25), whereas altered lipid metabolism contributes to energy deficiencies in diabetic cardiomyopathy (26). These results demonstrate the structural and functional functions of MAMs in preserving cellular homeostasis and point to the potential therapeutic benefits of these molecules in the treatment of cardiovascular disorders.

### 2.1 Ca^2+^ transport

Ca^2+^ are essential for excitation-contraction (EC) coupling in cardiac myocytes, where they directly contribute to energy production, contractile activation, and cell survival (27). Ca^2+^ enters the cell during an action potential through the activation of L-type calcium channels, which in turn initiates calcium release from the sarcoplasmic reticulum (SR) via ryanodine receptors 2 (RyR2) (4). Ca^2+^ is released and binds to contractile proteins, which in turn triggers muscle contraction. The sarcoplasmic/endoplasmic reticulum calcium ATPase (SERCA) either returns Ca^2+^ to the SR during relaxation or is discharged from the cell through the sodium-calcium exchanger (4). MAMs regulate Ca^2+^ transfer at the ER-mitochondria interface to ensure the precision of EC coupling, satisfy the high energy demands of cardiac myocytes, and maintain calcium homeostasis. Several essential proteins and regulatory mechanisms are involved in the transfer of Ca^2+^ at this interface. To begin, the ER membrane's inositol 1,4,5-trisphosphate receptors (IP3Rs) release Ca^2+^ in response to cellular signaling, thereby establishing a local high-calcium microdomain (28). The Ca^2+^ subsequently passes through voltage-dependent anion channels (VDACs) on the outer mitochondrial membrane and enters the mitochondrial matrix through the mitochondrial calcium uniporter (MCU) on the inner membrane, thereby supporting ATP production (29).

Key proteins regulating Ca^2+^ transfer at MAMs include the chaperone glucose-regulated protein 75 (Grp75) (Supplementary Figure S1), which bridges IP3Rs and VDACs to facilitate efficient Ca^2+^ transfer (28). The Sigma-1 receptor (Sig-1R), which enhances Ca^2+^ flux under ER stress by interacting with IP3Rs (30). Mitofusin-2 (MFN2) is another key protein that stabilizes ER-mitochondria contact sites, thereby maintaining calcium dynamics and ensuring efficient Ca^2+^ signaling between these organelles (31). Redox proteins such as Endoplasmic Reticulum Oxidoreductin 1 alpha (Ero1α) and Endoplasmic Reticulum Protein 44 (ERp44) modulate Ca^2+^ transfer by adjusting the redox state of IP3Rs (32–34), while selenoprotein N1 (SEPN1) (35) and Protein Tyrosine Phosphatase Interacting Protein 51 (PTPIP51) [interacting with ER protein Vesicle-Associated Membrane Protein-Associated Protein B (VAPB)] (36) help regulate ER-mitochondria Ca^2+^ transfer to prevent excessive flux. Additional proteins, such as calnexin (CNX) and Thioredoxin-related Transmembrane Protein 1, which modulate the redox state of SERCA2b, ensure proper ER Ca^2+^ levels, thereby supporting mitochondrial oxidative phosphorylation (37, 38). Glycogen Synthase Kinase 3 Beta (Gsk-3β), a kinase involved in mitochondrial apoptosis regulation, can specifically interact with and regulate the protein composition of the IP3R Ca^2+^ channeling complex (39). FUN14 Domain Containing 1 (FUNDC1), an autophagy receptor at MAMs, interacts with IP3Rs and other calcium-related proteins to maintain calcium homeostasis, protecting cardiac myocytes from ischemic damage (40). Cyclophilin D, a mitochondrial chaperone protein, interacts with the VDAC1/Grp75/IP3R1 complex to regulate Ca^2+^ transfer and mitochondrial dysfunction under hypoxia-reoxygenation stress (41).

In conclusion, these proteins form a highly integrated network at MAMs that ensures efficient Ca^2+^ transfer and signaling. By coordinating calcium signaling, MAMs support mitochondrial energy production to meet the workload demands of cardiac myocytes.

### 2.2 Lipid synthesis and transport

Some major proteins found in MAMs regulate the metabolism of phospholipids, cholesterol, and sphingolipids, all of which are essential for mitochondrial membrane integrity and cell function. Among the lipids generated at MAMs, phospholipids such as phosphatidylserine (PS) and phosphatidylethanolamine (PE) are necessary for mitochondrial membrane dynamics, whereas phosphatidylcholine (PC) is an important structural component of the cellular membrane (42, 43). Neutral lipids, such as triacylglycerol and cholesteryl esters, are critical for energy storage, membrane fluidity, and resistance to lipotoxicity and oxidative stress (44). The synthesis of these lipids is facilitated by various enzymes found in MAMs (Supplementary Figure S1).

Phosphatidylserine (PS) is produced by phosphatidylserine synthases (PSS1 and PSS2) on MAMs, transported to mitochondria via the Oxysterol-Binding Protein-Related Protein 5/8 (ORP5/8)-PTPIP51 complex, and converted to phosphatidylethanolamine (PE) by phosphatidylserine decarboxylase (45–47). PE maintains mitochondrial membrane curvature and function (48). Furthermore, phosphatidylethanolamine N-methyltransferase 2 can methylate PE to produce PC, thus completing a critical lipid production cycle (49). Diacylglycerol O-Acyltransferase 2 (DGAT2) catalyzes the formation of neutral lipids such as triglycerides (50). Acetyl-CoA cholesterol acyltransferase 1 (ACAT1) converts cholesterol into cholesteryl esters, hence regulating cholesterol levels (51). Fatty acyl-CoA ligase 4 (FACL4) converts free fatty acids into complex lipids needed for membrane formation and energy storage (52). The interaction of the Steroidogenic Acute Regulatory Protein (StAR) and Voltage-Dependent Anion Channel 2 (VDAC2) at MAMs promotes cholesterol transport into mitochondria, regulating the rate of steroid hormone synthesis and, as a result, cellular lipid balance and function (53).

Lipid transport between the ER and mitochondria is critical for cellular homeostasis and is mediated by proteins such as ATPase family AAA domain-containing protein 3 (ATAD3), which improves structural stability at the ER -mitochondria interface, promotes lipid transport, and ensures the efficient bidirectional exchange required for membrane and energy homeostasis (54). Caveolin-1 has a structural role in MAMs and can regulate membrane curvature and lipid signaling (55). Dysregulation of lipid metabolism at MAMs has a significant impact on cardiovascular pathology. Impaired phospholipid metabolism alters the structure of the mitochondrial membrane, reduces the efficiency of oxidative phosphorylation, and increases susceptibility to oxidative stress, all of which are associated with the onset of cardiovascular diseases (CVDs) (56). Abnormal cholesterol metabolism via ACAT1 causes lipid buildup, which leads to atherosclerosis and endothelial dysfunction (57). Similarly, DGAT2-mediated triglyceride production dysregulation may enhance cardiac steatosis and lipotoxic cardiomyocyte death (58). Dysfunctional lipid transport systems, such as decreased PS transfer via ORP5 or disturbed ER-mitochondrion connections via ATAD3, can affect calcium homeostasis, activate inflammasomes, and cause chronic inflammation. These mechanisms together hasten the progression of CVDs, such as atherosclerosis, diabetic cardiomyopathy, and heart failure (59).

### 2.3 Mitochondrial dynamics

Mitochondrial dynamics is the ongoing cycle of mitochondrial fusion and fission (60). Under physiological conditions, these processes enable mitochondria to constantly change their morphology, shape, and quantity in response to numerous cellular stimuli (61). The dynamic balance between fusion and fission is crucial for maintaining mitochondrial function, cellular health, and energy metabolism, especially in the heart, which has extremely high energy requirements (62).

MAMs play an important role in mitochondrial fission via proteins such as Dynamin-related protein 1 (Drp1), Fission 1 (Fis1), Mitochondrial Fission Factor (Mff), and FUNDC1 (Supplementary Figure S1). Mitochondrial fission begins with the recruitment of Drp1, a major Guanosine Triphosphatase (GTPase), to OMM (63). Several important proteins located at the MAMs, such as mitochondria Fis1, Mff, and mitochondrial dynamics proteins MiD49 and MiD5170, need to work in concert to facilitate this recruitment (64). These proteins cooperate to localize Drp1 at the ER-mitochondria interface, where it oligomerizes and activates its GTPase activity, causing mitochondrial membrane constriction and ultimately resulting in fission. Inverted formin 2 (INF2) is crucial for actin polymerization at MAMs, promoting actin-dependent mitochondrial constriction, Drp1 recruitment, and the increase in mitochondrial calcium levels required for division (65). Ras-related protein Rab-32 promotes Drp1 localization and the assembly of fission machinery at mitochondria (65). Under hypoxic conditions, the OMM protein FUNDC1 accumulates at MAMs and acts as a new Drp1 receptor, binding to the ER-localized protein CNX and facilitating fission (16).

Mitochondrial fusion enables the exchange of contents by integrating the outer and inner membranes, maintaining mitochondrial integrity and function (66). Fusion starts with MFN2 anchoring and merging the outer membrane, followed by Optic Atrophy Protein 1 (OPA1) regulating the inner membrane fusion (67). These stages help to exchange mitochondrial DNA, proteins, and metabolites, which is critical for maintaining mitochondrial integrity and adjusting to cellular energy demands (66). Interestingly, FUNDC1 also appears to play a role in regulating mitochondrial fusion. In response to low oxygen conditions (hypoxia), FUNDC1 facilitates mitochondrial fusion by preventing excessive fission, thus helping cells adapt to metabolic stress (68). MFN2 is found in MAMs, where it tethers mitochondria to the ER and other surrounding organelles by interacting with Mitofusin 1 (MFN1) or another MFN2 and promoting outer membrane fusion (69). OPA1 is located in the mitochondrial inner membrane, where it regulates inner membrane fusion and organizes cristae structures, which are essential for efficient ATP generation and stress response (70). Furthermore, the Endoplasmic Reticulum-Associated Degradation indirectly promotes mitochondrial fusion by removing faulty fusion proteins, hence maintaining mitochondrial dynamics in balance (30). The relationship between fusion and fission emphasizes the importance of MAMs and their associated proteins in mitochondrial dynamics, which is strongly linked to cellular health and disease progression.

## 3 The role of MAMs in DCM

Given the crucial role of MAMs in regulating various cellular processes, it is essential to explore their contribution to the development of DCM. In this review, we aim to summarize the key role of MAMs in regulating biological processes such as calcium overload, mitochondrial homeostasis, inflammation, ER stress, apoptosis, autophagy, necrosis, and ferroptosis within the context of DCM pathology. Additionally, we will identify and discuss specific MAM-related proteins involved in these processes (Supplementary Figure S2).

### 3.1 Ca^2+^ overload

Mutations in the RNA Binding Motif Protein 20 gene lead to calcium overload in myocardial cells, resulting in elevated levels of Ca^2+^ in the SR (71). The calcium channel blocker verapamil has been shown to effectively alleviate arrhythmias in DCM patients (71), supporting the critical role of calcium dysregulation in the pathogenesis of DCM. Abnormal calcium signaling at MAMs can affect myocardial contractility and relaxation, promoting myocardial fibrosis and cardiac remodeling, which are central factors in the progression of DCM (72). Several key proteins located at MAMs undergo changes during the development of DCM.GSK-3β protein, present at MAMs, interacts with the IP3R complex to regulate calcium exchange (73) (Supplementary Figure S2). Studies have shown that Coxsackievirus B3 infection activates GSK-3β, inducing myocardial injury and apoptosis, leading to DCM (74).

Additionally, calcium/calmodulin-dependent protein kinase II (CaMKII) regulates calcium transfer at MAMs through phosphorylation of the RYR2, and its overactivation is closely associated with DCM (Supplementary Figure S2). Elizabeth et al. (75) reported that CaMKII overexpression in mice leads to severe ATP depletion and SR calcium leakage in myocardial cells, resulting in advanced DCM. LMNA gene mutations cause LMNA-DCM, accounting for 4%–8% of all DCM cases (76). Research by Hang et al. (77) showed that iPSC-derived cardiomyocytes from LMNA-DCM patients exhibit abnormal calcium handling and elevated oxidative stress (ROS). ROS activation of the CaMKII-RyR2 pathway further exacerbates arrhythmias and nuclear membrane deformation (78). Dickkopf 3 ameliorates the development of familial dilated cardiomyopathy by downregulating CaMKII (78).

Another important regulator in calcium cycling is PLN, which controls calcium reuptake by regulating SERCA2, thereby affecting cardiac contraction and relaxation (Supplementary Figure S2). PLN mutations or dysfunction decrease SERCA2 activity, disrupting calcium cycling and accelerating DCM progression (79, 80). After LAVDs support, the significant dysregulation of SERCA2 in DCM patients was restored, further emphasizing the importance of SERCA2 in DCM pathophysiology (81). In Duchenne muscular dystrophy (DMD), mutations in the dystrophin gene not only affect skeletal muscle but also impact cardiac muscle, leading to cardiac dilation and eventual failure, progressing to DCM (82). Studies (83) have shown that in DMD mouse models, early cardiac damage is associated with elevated expression of IP3R1 and its regulatory subunit Sig-1R, enhancing the IP3R1-GRP75-VDAC-MCU complex and increasing mitochondrial calcium content. Metformin may help reverse these changes (84). In patients with DCM and doxorubicin (DOX)-treated mice, a downregulation of FUNDC1 was observed in cardiac tissues. This deficiency exacerbated DOX-induced cardiac damage and heart dysfunction in mice (85). The regulation of Ca^2+^ homeostasis mediated by MAMs plays a crucial role in maintaining normal myocardial function and cardiomyocyte activity.

### 3.2 Mitochondrial destabilization

Mitochondrial fusion and fission are key mechanisms in maintaining the dynamic balance of mitochondrial morphology and function and are crucial for cardiac homeostasis and normal cardiac remodeling processes. A large number of very small and fragmented mitochondria have been found in the myocardial tissues of end-stage DCM patients, which are associated with the loss of mitochondrial fusion/fission balance (86). Recent studies have suggested that mutations in fission genes may be a cause of DCM. Immediate postnatal gene ablation of Drp1 in mice is lethal (87). In adult Drp1 knockout mice, myocardial mitochondria become enlarged and elongated, with reduced volume. These mice develop replacement fibrosis, increased mitophagy, calcium uptake, superoxide production, and permeability transition pore opening, leading to cardiomyocyte apoptosis. They display typical DCM features, including ventricular wall thinning, dilation, and an increased r/h ratio (left ventricular end-diastolic diameter to wall thickness) (87–89). In models with impaired Drp1 function, upregulation of the fusion factors MFN2 and OPA1 provide some compensatory effects against mitochondrial damage (87). Moreover, in Mff gene-deficient mice, mitochondrial fission capability is significantly reduced, resulting in excessive mitochondrial fusion and network formation, ultimately leading to premature death due to severe DCM (90). However, researchers have found that regulating the expression of fusion-related proteins (such as MFN1 or OPA1) can improve this pathological damage (90). These findings suggest that the balance between mitochondrial fusion and fission is crucial for cardiomyocytes and provides new therapeutic perspectives. In the treatment of DCM caused by defects in fission genes, therapy may not only target fission, with drugs like LCZ696 (a novel angiotensin receptor neprilysin inhibitor) or Drp1-specific inhibitors like Midivi-1 (91), but also employ drugs that modulate fusion proteins (e.g., MFN2 agonists).

Of course, the lack of MFN1 and MFN2 genes is lethal in mouse embryos, while adult mice show mitochondrial fragmentation and respiratory chain dysfunction, leading to fatal DCM (92). A study of endomyocardial biopsies from the interventricular septum of 22 idiopathic dilated cardiomyopathy (IDCM) patients revealed that MFN1 expression was significantly reduced in the myocardium of treatment-resistant IDCM patients. Restoring MFN1 function or increasing its expression may be beneficial for treatment-resistant IDCM patients (93). In addition, abnormal processing of the fusion protein OPA1 leads to excessive mitochondrial fission and fragmentation, ultimately causing ventricular dilation and heart failure. Regulation of key proteases that affect OPA1 processing (such as Opa1 mitochondrial dynamin-like GTPase-1 and YME1 like 1) can significantly improve mitochondrial morphology and function in cardiomyocytes (94). In conclusion, changes in mitochondrial fusion and fission mechanisms in the heart promote mitochondrial metabolic damage, thereby triggeringDCM. The associated proteins are primarily located on MAMs (Supplementary Figure S2), especially MFN2, playing an important regulatory role in the distance between the ER and mitochondria.

### 3.3 Inflammation

Cardiac injury caused by genetic, immune, or infectious factors triggers inflammation and activates immune repair processes. However, sustained or excessive inflammatory responses can lead to fibrosis, oxidative stress, and mitochondrial dysfunction, which are major driving forces in the onset and progression of DCM (2). The NOD-like receptor protein 3 (NLRP3) inflammasome is currently the only inflammasome known to be localized on MAMs (95) (Supplementary Figure S2). On the one hand, its components, including NLRP3, Apoptosis-associated speck-like protein containing a CARD (ASC), and Caspase-1, aggregate and assemble at MAMs (96). On the other hand, MAMs dysfunction-induced calcium overload, oxidative stress, mitochondrial dysfunction (such as the release of ROS, mitochondrial DNA, cardiolipin, mitochondrial-associated antiviral signaling, and thioredoxin-interacting protein) can activate the NLRP3 inflammasome, amplifying inflammatory signaling and triggering downstream effects (97, 98).

Cheng et al. (99) conducted a study on 18 human cardiac tissue samples and found that, compared to patients without a history of heart disease, DCM patients exhibited ASC speck formation in cardiomyocytes, along with elevated protein expression of caspase-1 and the pro-inflammatory cytokines IL-1β and IL-18. Plasma levels of IL-1β and IL-18 were also significantly higher in DCM patients, indicating that the NLRP3 inflammasome is activated in DCM and is likely involved in promoting cardiac dysfunction (100, 101). Following its assembly, the NLRP3 inflammasome catalyzes the production of caspase-1, which cleaves Gasdermin D (GSDMD) to form its active N-terminal functional domain (GSDMD-NT) (102). This process facilitates pore formation in the cell membrane, ultimately inducing pyroptosis. In myocardial tissues from nine DCM patients, GSDMD-NT levels were significantly elevated (99).

Interestingly, the researchers also performed triple immunostaining for active caspase-1, TdT-mediated dUTP Nick-End Labeling, and α-actin on cardiac specimens and observed that pyroptotic cardiomyocytes were markedly more abundant than apoptotic ones in DCM. These findings suggest that NLRP3-mediated pyroptosis may play a critical role in the progression of DCM (99). Furthermore, dapagliflozin has been shown to inhibit the activation of the NLRP3 inflammasome by suppressing p38-dependent Toll-like receptor 4 expression and reducing ROS production, highlighting its potential therapeutic value in treating DCM (103). Additionally, the NLRP3 inflammasome has been found to induce myocardial fibrosis and impair myocardial contractility; however, its specific role in DCM remains to be elucidated (104, 105).

### 3.4 ER stress

Protein mutations, oxidative stress, ROS production, and aberrant intracellular Ca^2+^ handling can all increase the demand for protein synthesis, disrupting the ER homeostasis and leading to the accumulation of misfolded proteins within the ER lumen, thereby triggering ER stress (ERS) (106). In response, the cell activates three key sensors of the unfolded protein response (UPR): PKR-like ER kinase (PERK), activating transcription factor 6 (ATF6), and inositol-requiring enzyme 1α (IRE1α) (107). These sensors dissociate from the chaperone protein Binding Immunoglobulin Protein [Bip, also known as Glucose-Regulated Protein 78 (GRP78)] and initiate different signaling pathways to restore ER homeostasis and prevent cell death (107). Proteins such as PERK, IRE1α, and Bip are highly enriched in MAMs (108, 109) (Supplementary Figure S2), which also regulate ERS signaling. For instance, VAPB directly suppresses UPR by interacting with ATF6 (110). Dysfunction of MAMs can exacerbate or induce ERS.

Several studies have shown that in patients with DCM, levels of UPR markers—such as BiP, ATF6, phosphorylated eukaryotic initiation factor 2α (P-eIF2α), and X-box Binding Protein 1—are significantly elevated, indicating that ERS and UPR pathways are activated in the myocardium of DCM patients (81, 111). UPR can serve as a compensatory mechanism, protecting myocardial cells in the short term. For example, PERK activation leads to the phosphorylation of eIF2α, temporarily halting protein synthesis to restore ER balance and alleviate ER stress (112, 113). Furthermore, PERK maintains the expression of SERCA2a, regulating calcium homeostasis in cardiomyocytes, preventing ER stress, and offering protection in heart failure (114–116). However, prolonged activation of the UPR (referred to as maladaptive UPR) can impair cellular homeostasis, triggering autophagy or apoptosis, and further exacerbating cardiac structural and functional damage (117–119). As an important site for calcium signaling and ROS regulation, MAMs play a significant role in amplifying the ER stress response. Under excessive ER stress, IRE1α interacts with the adaptor proteins TNF receptor-associated factor 2 and apoptosis signal-regulating kinase 1 to activate downstream apoptotic signaling (120). Additionally, IRE1α regulates IP3Rs, inducing calcium dysregulation and further promoting apoptosis (121). The accumulation of PERK in MAMs not only activates protective signaling via the eIF2α activating transcription factor 4 (ATF4) pathway but also promotes the expression of pro-apoptotic factors such as C/EBP homologous protein (CHOP), exacerbating cardiomyocyte death under prolonged stress (122). Compounds such as Ferulic Acid, Pterostilbene, and Tyrosol have been shown to alleviate ER stress-induced cardiac injury, likely through their modulation of MAMs. These compounds may reduce the activation of the PERK/eIF2α/ATF4/CHOP pathway, thereby decreasing cardiomyocyte apoptosis and protecting the heart from ER stress-mediated damage. This highlights the therapeutic potential of targeting MAMs in combating ER stress-induced cardiac injury (123).

### 3.5 Apoptosis

The release of cytochrome c (Cytc) from mitochondria and the activation of caspase-3 in the myocardial tissue of end-stage DCM patients provide molecular evidence for the involvement of apoptosis in the development of DCM (124, 125). MAMs typically regulate apoptosis by modulating intracellular oxidative stress, mitochondrial function, Ca^2+^ concentration, and ER stress. Dysregulated expression of Ero1α and SHC Adaptor Protein p66 (p66Shc) on MAMs can directly promote the generation of ROS (Supplementary Figure S2), leading to oxidative stress in cardiomyocytes and triggering cardiomyocyte apoptosis (126, 127). In DCM, factors such as DNA damage and myocardial mechanical stretch increase the expression of Tumor Protein p53 (p53), which accumulates in MAMs and promotes apoptosis through multiple molecular mechanisms (128–130). p53 directly activates the pro-apoptotic gene Bcl-2-associated X protein (Bax), leading to Bax translocation to the outer mitochondrial membrane, where it interacts with the anti-apoptotic protein B-cell lymphoma 2 (Bcl-2), which also relocalizes to MAMs (131, 132). This interaction disrupts the mitochondrial outer membrane integrity, facilitating Cytc release from the mitochondria. Cytc then activates caspase 9, which further triggers downstream caspase activation (caspase 3 and caspase 7), leading to the cleavage of critical cellular proteins and ultimately leading to cardiomyocyte apoptosis (129, 130, 133, 134). Interestingly, although the anti-apoptotic factor Bcl-2 levels increase in DCM, this increase is insufficient to counterbalance the pro-apoptotic signals, suggesting a compensatory mechanism that fails under prolonged stress (133, 135). Moreover, studies have shown that under stress conditions, p53 can also bind to SERCA2, leading to mitochondrial Ca^2+^ overload and apoptosis (128). However, when the activity of cardiac SERCA2 ATPase is reduced, p53 takes on a protective role (136). The modulation of these key pathways, including the Bcl-2 family, Bax, and the caspase cascade, within MAMs underscores the critical role of MAM dysfunction in exacerbating apoptosis in DCM. The mitochondrial fusion protein OPA1 on MAMs can directly participate in cytochrome c release by affecting the opening of crista junctions, located at the junction of mitochondrial cristae and the boundary membrane, thus regulating apoptosis in the progression of DCM (137, 138). Protein Kinase B (PKB, also known as Akt) on MAMs can phosphorylate IP3R3, reducing the sensitivity of cells to Ca^2+^-dependent apoptosis (139, 140). The functional loss of its upstream regulator, mechanistic Target of Rapamycin Complex 2 (mTORC2), decreases Akt activity and accelerates cardiomyocyte apoptosis (141, 142). mTORC2 is also localized to MAMs (139). These studies suggest that MAMs play a critical role in cardiomyocyte apoptosis during the progression of DCM.

### 3.6 Autophagy

Autophagy is a key cellular mechanism that helps cells respond to stress by degrading damaged cellular structures, particularly under conditions of ER stress and excessive ROS production, thereby aiding in the restoration of cellular homeostasis; however, excessive autophagy can also lead to cardiomyocyte injury and death (143–145). Although numerous studies have identified abnormal autophagy in DCM, with abundant autophagic vacuoles (autophagosomes and autolysosomes) and lysosomes in myocardial cells, the general conclusion suggests that autophagy plays a protective role in cardiomyocytes, reducing myocardial degeneration and reversing ventricular remodeling (146–148). Some studies indicate that many key proteins directly involved in autophagy, such as Parkin E3 ubiquitin ligase (Parkin), Beclin 1 (BECN1), and AMP-activated protein kinase (AMPK), are localized to MAMs, where autophagosomes are formed (149–151) (Supplementary Figure S2).

Parkin deficiency affects the ubiquitination of MFN2, disrupting the integrity of MAMs, inhibiting autophagy, and leading to mitochondrial dysfunction, which in turn triggers severe DCM (152, 153). Bcl-2 binds to BECN1 to suppress autophagy, but during cellular stress, BECN1 dissociates from Bcl-2, promoting autophagy (154). Researchers have observed that activation of Macrophage Stimulating 1 in DCM strengthens the binding of Bcl-2 to BECN1, thereby inhibiting autophagy and promoting apoptosis (155). AMPK, as an energy sensor, regulates the dynamic stability of MAMs in cardiomyocytes by phosphorylating MFN2 and activating autophagy under energy stress conditions (156). MAMs also influence autophagy through the regulation of mitochondrial function, calcium ion transport, and lipid synthesis (149). Drp1 promotes autophagy by regulating mitochondrial fission, and its dysfunction (such as resistance to oligomer disassembly) leads to mitochondrial autophagy impairment, reduced calcium uptake, and compromised ATP synthesis, thereby triggering DCM (157). PE, a marker of autophagosomal membrane expansion, is a major receptor for Autophagy-Related Gene 8 (ATG8), and in DCM studies, ATG8 is often used as a marker of autophagic activity (158). However, how MAMs regulate the relationship between PE and ATG8 to influence autophagy requires further exploration. Ca^2+^ promote autophagy by inhibiting the mechanistic target of the rapamycin (mTOR) pathway via AMPK and CaMKII (159–161). However, excessively high calcium concentrations can activate calpains and calcineurin, which negatively regulate autophagy, potentially impairing myocardial function and contributing to the development of DCM (159–161). Tacrolimus and other rapamycin analogs can restore autophagy by blocking mechanistic Target of Rapamycin Complex 1 activity, preventing the progression of LMNA mutation-induced cardiomyopathy (162). In summary, MAMs regulate autophagy through multiple pathways, including mitochondrial fusion and fission, lipid metabolism, and calcium homeostasis.

### 3.7 Necroptosis

During the transition from pressure-overload hypertrophy to DCM, the mitochondria-targeted pro-apoptotic Bcl-2 family protein Nix (Nip3-like protein X) induces cardiomyocyte apoptosis by promoting the oligomerization of Bax and Bcl-2 homologous antagonist/killer (163). On the other hand, endoplasmic/sarcoplasmic reticulum (ER/SR)-targeted Nix increases ER/SR calcium storage and triggers mitochondrial permeability transition pore (MPTP) opening in cardiomyocytes via MAMs. This leads to ATP depletion, mitochondrial swelling, OMM rupture, and Cytc release, resulting in a form of programmed cell death distinct from apoptosis, known as necroptosis (164–166) (Supplementary Figure S2). The cardiotoxicity of the anti-cancer drug sorafenib may cause DCM, with its mechanism potentially linked to increased MAMs formation, reduced distance between MAMs and mitochondria, and consequent Ca^2+^ overload in cardiomyocytes (167). This overload activates CaMKII and the Receptor Interacting Protein Kinase 3 Mixed Lineage Kinase Domain-Like Protein cascade, inducing necroptosis. Overexpression of MFN2 shows promise in mitigating sorafenib-induced cardiac dysfunction (167).

### 3.8 Ferroptosis

Ferroptosis is a novel iron-dependent form of cell death, whose mechanism involves the generation of free radicals via the Fenton reaction, lipid peroxidation, and the inactivation of intracellular antioxidant systems, such as the depletion of glutathione (GSH) and glutathione peroxidase 4 (GPX4) (168). DOX, gene knockout of ferritin H, and iron deposition induced by various factors can directly trigger ferroptosis in cardiomyocytes, leading to cardiac dysfunction and heart injury (169–171). This suggests that ferroptosis is an important pathological factor in cardiomyopathy. However, evidence regarding the role of ferroptosis in DCM remains limited, with much of the research still in the bioinformatics phase. Studies have shown that disruptions in Ca^2+^ homeostasis at the MAMs, accumulation of unsaturated fatty acids, and the buildup of ROS can all induce ferroptosis (172–174). Heme Oxygenase 1 enrichment at MAMs plays a critical role in the pathogenesis of DCM by regulating iron homeostasis (175). In a DOX-induced mouse model, overactivation of the Nrf2-HMOX1 axis leads to HMOX1-mediated heme degradation and the release of free iron (Fe^2+^), which accumulates in mitochondria (169). This iron overload generates ROS through the Fenton reaction, triggering oxidative stress, lipid peroxidation, and ferroptosis, thereby exacerbating myocardial injury and dysfunction. Inhibition of HMOX1 activity by zinc protoporphyrin IX reduces free iron release, significantly alleviating myocardial damage and dysfunction (169). Targeting HMOX1 or MAMs function may provide new therapeutic strategies for DCM (Supplementary Figure S2). In DOX-induced cardiomyopathy, the FUN14 domain containing 2 (FUNDC2) protein at the OMM regulates mitochondrial GSH levels by influencing the stability of the mitochondrial glutathione transporter Solute Carrier Family 25 Member 11 (SLC25A11) and GPX4, thereby promoting ferroptosis (176). Wang et al. (177) through microarray data analysis, identified the signal transducer and activator of transcription 3 (STAT3) as a gene associated with ferroptosis in DCM, and the transcription factor STAT3 encoded by this gene is located in the MAMs (178).

## 4 Discussion

This article delves into the critical role of MAMs in the pathogenesis and progression of DCM. MAMs play an essential role in maintaining cardiac cell function by precisely regulating intracellular calcium homeostasis, lipid metabolism, and mitochondrial dynamics. Dysregulation of MAMs leads to calcium overload, mitochondrial dysfunction, and activation of cell death pathways such as autophagy, apoptosis, necroptosis, ferroptosis, and necrosis, all of which are closely associated with the clinical and pathological features of DCM (Supplementary Table S1). These findings highlight MAMs as a key node in cellular dysfunction and pathological transitions. Modulating the function of MAMs may not only provide early biomarkers for cardiac diseases but also offer new perspectives and potential targets for therapeutic interventions and the development of novel treatment strategies for DCM. However, most experimental studies on MAMs have focused on cardiomyocytes, with relatively fewer studies on fibroblasts and endothelial cells. Further research is needed to explore whether MAMs in different types of cardiac cells have distinct effects on the onset and progression of DCM. Additionally, as indicated by previous analyses, the protein expression profile of MAMs in DCM caused by different genetic mutations often varies, and the roles of some proteins exhibit a dual nature. Therefore, finding a balance between the protective effects and potential risks of MAMs is crucial. It is particularly important to develop more precise intervention strategies tailored to different genetic types of DCM. With the ongoing in-depth investigation of MAMs as a therapeutic target, we anticipate that this will lead to groundbreaking advances in the clinical treatment of DCM, improve patient outcomes, and provide new strategies for comprehensive cardiac disease management.
