# Supplementary material for: Mitochondria associated membranes in dilated cardiomyopathy: connecting pathogenesis and cellular dysfunction
Source: Front Cardiovasc Med. 2025 Mar 17;12:1571998. doi: 10.3389/fcvm.2025.1571998 (PMC11955654; doi:10.3389/fcvm.2025.1571998)
Supplement: Supplementary file 1 [file Datasheet1.pdf]

Supplementary Material

1 Supplementary Figures

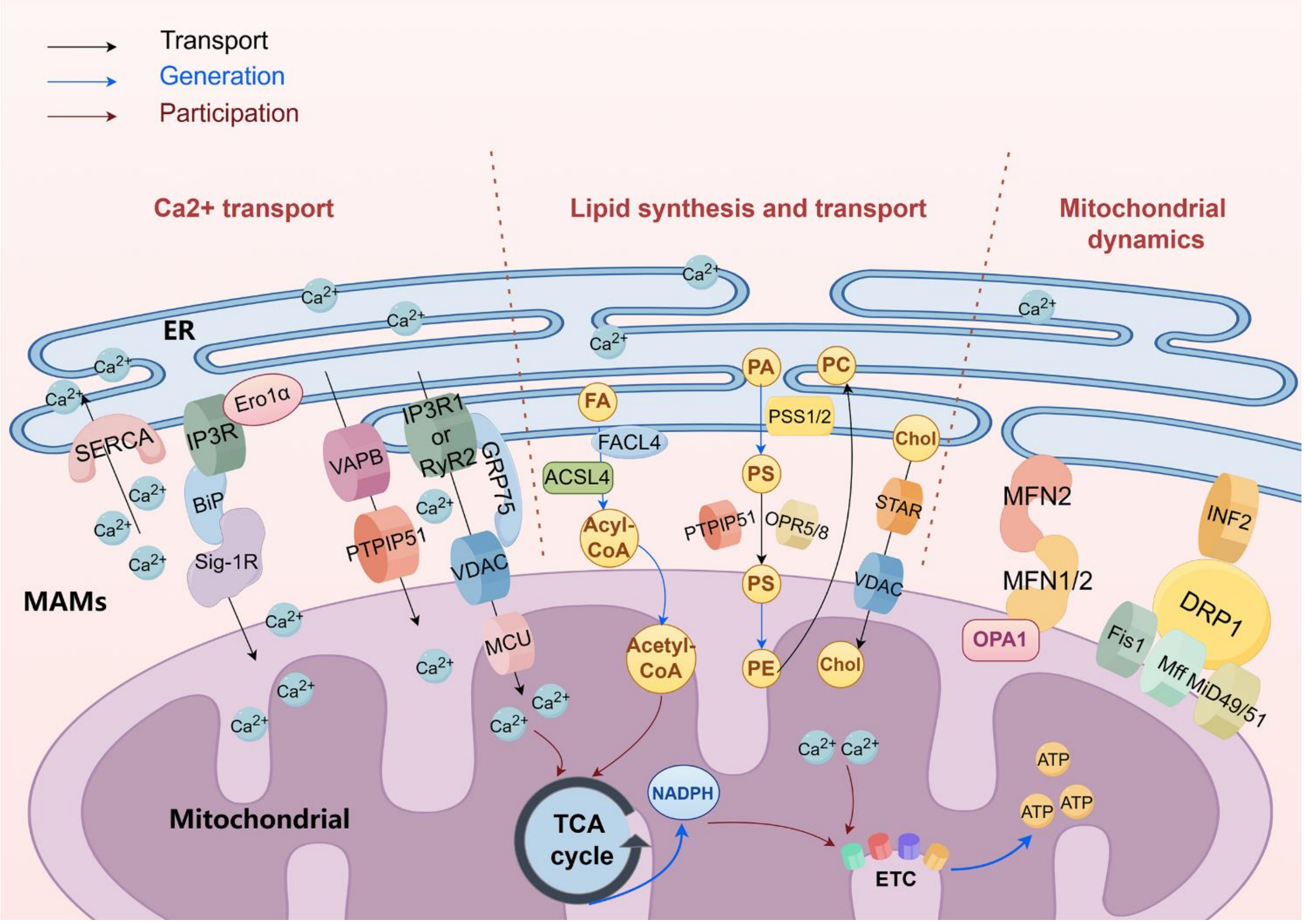

Supplementary Figure 1. Functional roles of MAMs. MAMs play regulatory roles in Calcium Transport, Lipid Metabolism, and Mitochondrial Dynamics The schematic illustrates the roles of key proteins and molecules involved in calcium transport, lipid synthesis and transport, mitochondrial dynamics, and energy metabolism at the Mitochondria-associated membranes (MAMs). In the calcium transport section, proteins such as SERCA pump Ca^{2+} into the ER, while IP3R and RyR2 release Ca^{2+} into the MAMs, where Sig-1R, BiP, and Ero1α regulate their stability and function. PTPIP51, VAPB, GRP75, VDAC, and MCU mediate Ca^{2+} transport into the mitochondria. In the lipid synthesis and transport section, fatty acids (FA) are activated by ACSL4 and FACL4 to form Acyl-CoA. Lipid intermediates such as PA and PS are synthesized by PSS1/2, transferred to the mitochondria via ORP5/8, and converted into PE or PC. Cholesterol (Cho) is transported by STAR to mitochondria for membrane stabilization. The mitochondrial dynamics section highlights proteins such as MFN1/2 and OPA1, which mediate mitochondrial fusion, while DRP1, Fis1, INF2, Mff, and MiD49/51 regulate mitochondrial fission. Lastly, energy metabolism is depicted through the TCA cycle and ETC, located in the mitochondrial matrix and inner membrane, respectively. These processes generate ATP while producing ROS as byproducts. The integration of Ca^{2+}, lipids, and mitochondrial dynamics highlights the essential roles of MAMs in cellular function.

Arrow Color Key: Black arrows (Transport): Represent the transfer of molecules or ions between organelles or membranes, such as calcium ions (Ca^{2+}) and lipid intermediates. Blue arrows (Generation): Indicate the synthesis or formation of molecules, such as lipid intermediates and metabolic products. Brown arrows (Participation): Depict proteins or molecules actively participating in or contributing to a specific process or function.

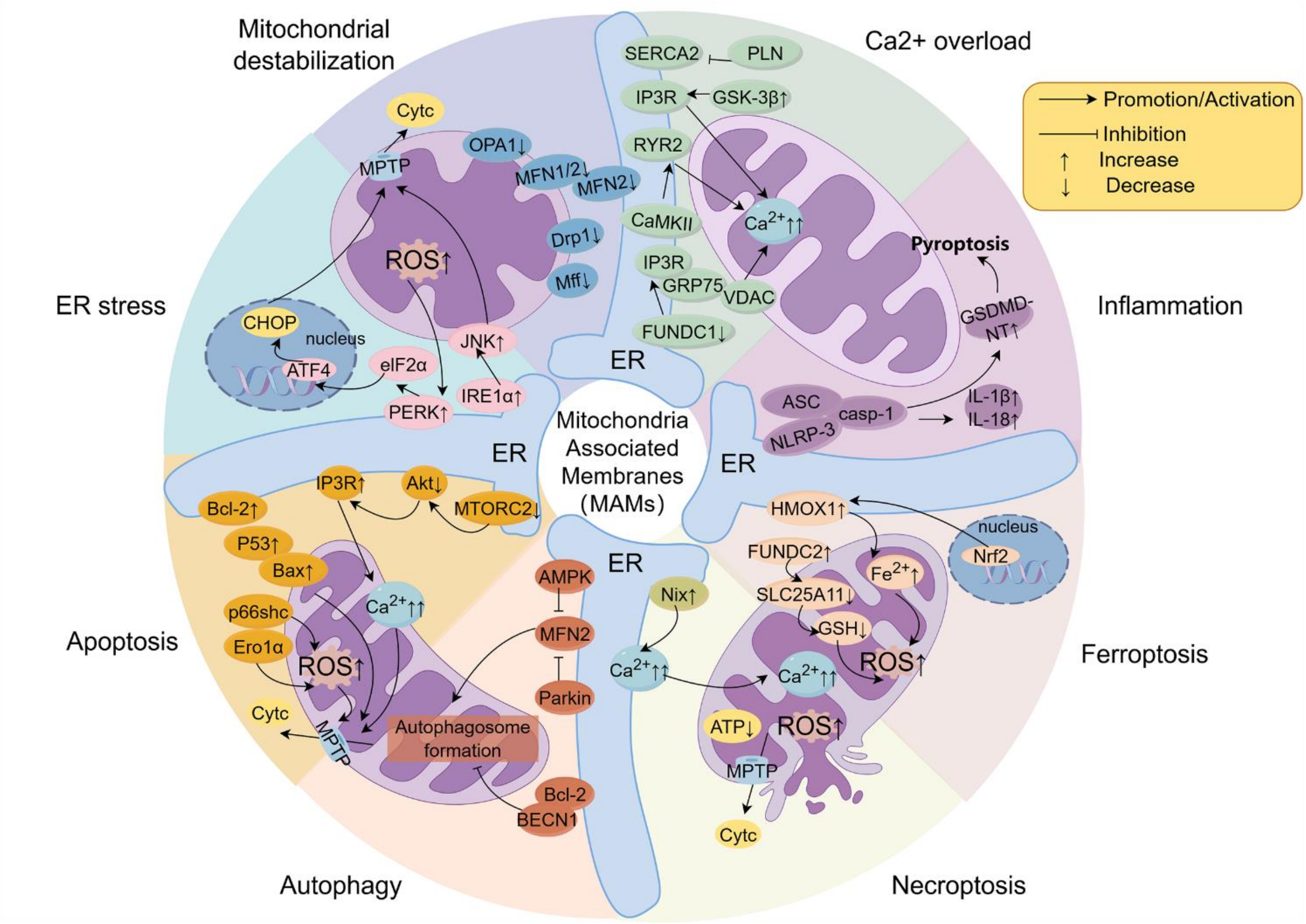

Supplementary Figure 2. The Role of MAMs in DCM The role of MAMs in dilated cardiomyopathy is illustrated in the figure with each of the eight functional subdivisions, with straight arrows indicating promotion, inhibitory arrows indicating inhibition, and upward arrows indicating increase and downward arrows indicating decrease. **Ca^{2+} overload:** MAM dysfunction in DCM leads to a dysregulation of calcium homeostasis, contributing to Ca^{2+} overload. PLN inhibits SERCA2, impairing calcium reuptake into the sarcoplasmic reticulum and elevating cytosolic calcium levels. Elevated GSK-3β enhances IP3R activity, further promoting calcium release from the endoplasmic reticulum. Additionally, CaMKII activates RYR2, amplifying calcium release from the sarcoplasmic reticulum. The IP3R-GRP75-VDAC complex facilitates increased calcium transfer into mitochondria, exacerbating mitochondrial calcium overload. Furthermore, reduced FUNDC1 expression enhances IP3R function, promoting further calcium release. **Mitochondrial destabilization:** Mitochondrial destabilization in DCM is driven by the downregulation of key mitochondrial fusion and fission proteins, leading to impaired mitochondrial dynamics. The decreased expression of OPA1 impairs mitochondrial fusion, promoting fragmentation. Additionally, the downregulation of MFN2 reduces MFN1/2 complex formation, further inhibiting mitochondrial fusion and contributing to mitochondrial instability. The reduced levels of Drp1 and Mff disrupt

mitochondrial fission, exacerbating mitochondrial fragmentation. These alterations in mitochondrial dynamics lead to impaired energy production, increased mitochondrial permeability, and enhanced ROS production, further destabilizing mitochondria and promoting cellular dysfunction. **ER stress:** In DCM, ER stress is initiated by the upregulation of IRE1α, which activates the JNK pathway, promoting mitochondrial dysfunction and the release of cytochrome c via MPTP opening. Increased ROS levels further activate the PERK signaling pathway, leading to phosphorylation of eIF2α. This event stimulates the transcription of ATF4 in the nucleus, which then upregulates CHOP, a key protein involved in apoptosis. The elevated CHOP expression exacerbates mitochondrial dysfunction by enhancing MPTP opening and cytochrome c release, ultimately driving apoptotic cell death. **Apoptosis**: Apoptosis in DCM is driven by multiple interconnected signaling pathways. The downregulation of mTORC2 leads to reduced Akt activity, which increases the expression of IP3R, promoting excessive calcium release and elevating cytosolic calcium levels. Elevated p53 expression activates pro-apoptotic pathways, increasing the levels of Bax, which promotes mitochondrial outer membrane permeabilization. Although the anti-apoptotic protein Bcl-2 is compensatorily upregulated, it is insufficient to counteract the apoptotic signals. Additionally, p66shc and Ero1α induce ROS production, which further amplifies mitochondrial dysfunction. These pathways collectively converge on the opening of the MPTP, leading to the release of cytochrome c from mitochondria, thereby triggering the apoptotic cascade. **Autophagy**:Autophagy regulation in DCM is influenced by several key proteins. Bcl-2 interacts with BECN1, preventing the formation of autophagosomes and inhibiting autophagy initiation. Both AMPK and Parkin suppress the expression of MFN2, thereby impairing mitochondrial dynamics and disrupting the formation of autophagic vesicles. The dysfunction in these regulatory pathways leads to defective autophagy, resulting in the accumulation of damaged mitochondria and other cellular components, contributing to cellular stress and dysfunction. **Necroptosis:** In DCM, Nix upregulation enhances ER calcium storage, which subsequently increases calcium transfer to mitochondria. This elevation in mitochondrial calcium triggers the opening of the MPTP, initiating a cascade of mitochondrial dysfunction. The opening of the MPTP leads to ATP depletion, mitochondrial swelling, and outer mitochondrial membrane (OMM) rupture. **Ferroptosis:** In DCM, Nrf2 activation drives the transcription of HMOX1, which promotes the release of iron from intracellular stores. This increase in iron levels may contribute to the initiation of ferroptosis by facilitating lipid peroxidation. Additionally, the upregulation of FUNDC2 leads to the downregulation of SLC25A11, reducing glutathione levels and impairing antioxidant defense. This imbalance, coupled with elevated ROS levels, further promotes ferroptosis through oxidative stress, driving iron-dependent cell death. **Inflammation:** In DCM, the assembly of the NLRP3 inflammasome, consisting of ASC, NLRP3, and caspase-1, triggers the upregulation of pro-inflammatory cytokines IL-1β and IL-18. The activation of caspase-1 leads to the cleavage of gasdermin D (GSDMD), which facilitates the formation of membrane pores. This pore formation disrupts the cell membrane, inducing pyroptosis, a form of inflammatory cell death that exacerbates the inflammatory response and contributes to tissue damage in the heart.

2 Supplementary Table

Table 1 Components of MAMs involved in DCM

Category	Proteins	Subcellular Localization	Function in MAMs	Role in DCM
**Calcium Tran sport**	GSK-3β	MAMs, Cyto plasm	Interacts with IP3R com plex to regulate calcium exchange; downstream of Akt signaling.	Heterozygous knockout of GSK-3 β inhibits cardiac dilation and fibr osis, but complete deletion causes catastrophic cardiomyocyte mitosi

				s. Potential target for novel pharm acological inhibitors.
	CaMKII	MAMs, Cyto plasm	Phosphorylates calcium -handling proteins (e.g., RyR2), influencing calc ium release.	Overexpression of CaMKII is clos ely associated with DCM; inhibitin g CaMKII overexpression can pre vent or reverse some forms of acq uired DCM.
	PLN	SR, MAMs	Modulates SERCA2 act ivity to regulate calcium uptake at MAMs.	Mutations or dysfunction of PLN l ead to reduced SERCA2 activity, a ccelerating DCM progression; incr easing SERCA2/PLN ratio can im prove cardiac function.
	Sig-1R	ER membran e, MAMs	Binds to IP3R1 to regul ate calcium signaling.	Elevated Sig-1R expression in DM D mouse models is associated with increased mitochondrial calcium; Metformin may reverse these chan ges.
	IP3R1-G RP75-VD AC	ER membran e, Mitochond rial outer me mbrane, MA Ms	GRP75 bridges IP3R an d VDAC1, facilitating c alcium transfer from ER to mitochondria.	Enhanced IP3R1-GRP75-VDAC c omplex in DMD models increases mitochondrial calcium; Metformin may reverse these changes.
	FUNDC1	Mitochondria l outer memb rane, MAMs	Regulates MAM-mediat ed calcium homeostasis.	Downregulation of FUNDC1 in D CM patients exacerbates DOX-ind uced cardiac damage; restoring FU NDC1 function may improve cardi ac dysfunction.
**Mitochondria l Dynamics**	Drp1	Mitochondria l outer memb rane, Cytopla sm	Regulates mitochondrial fission to maintain mito chondrial morphology.	Impaired Drp1 function causes exc essive mitochondrial fusion and ca rdiomyocyte apoptosis, leading to DCM; Drp1 inhibitors (e.g., Midiv i-1) or fusion regulators (e.g., Mfn 2) may have therapeutic potential.
	Mff	Mitochondria l outer memb rane	Promotes mitochondrial fission.	Mff deficiency leads to excessive mitochondrial fusion and DCM; re gulating fusion proteins (e.g., Mfn 1, OPA1) may improve pathologic al damage.
	Mfn1	Mitochondria l outer memb rane, MAMs	Regulates mitochondrial fusion and maintains m itochondrial network.	Mfn1 expression is decreased in I DC patients; restoring its function may benefit IDC patients unrespon sive to treatment.
	Mfn2	Mitochondria l outer memb rane, MAMs	Regulates mitochondrial fusion and adjusts ER-mitochondria distance.	Mfn2 loss leads to mitochondrial f ragmentation and DCM; Mfn2 ago nists may have therapeutic potenti al.
	OPA1	Mitochondria l inner memb rane, MAMs	Regulates mitochondrial fusion and cristae struct ure.	Abnormal OPA1 processing cause s excessive mitochondrial fission a nd DCM; regulating key proteases

				(e.g., OMA1, YME1L) can improve cardiomyocyte function.
**Inflammation**	NLRP3	MAMs, Cytoplasm	Assembles at MAMs to activate inflammatory signaling.	NLRP3 inflammasome activation is associated with DCM; Dapagliflozin inhibits NLRP3 activation, showing potential for DCM treatment.
**ER Stress**	BiP	ER membrane, MAMs	Acts as a molecular chaperone to regulate UPR signaling.	Elevated BiP levels in DCM patients indicate activated ER stress and UPR pathways; BiP provides short-term protection, but prolonged activation may lead to cell death.
	IRE1α	ER membrane, MAMs	Activates apoptotic signals through TRAF2 and ASK1, regulates IP3R to disrupt calcium homeostasis.	IRE1α activation exacerbates cardiomyocyte apoptosis; inhibiting IRE1α may reduce DCM progression.
	PERK	ER membrane, MAMs	Phosphorylates eIF2α to reduce protein synthesis and maintain SERCA2a expression.	PERK activation protects cardiomyocytes short-term, but chronic activation may lead to cell death; drugs like AICAR can reduce ER stress by inhibiting PERK.
**Apoptosis**	P53	MAMs, Nucleus	Upregulates Bax to promote cytochrome c release from mitochondria, activating caspase cascade.	Increased P53 expression is linked to cardiomyocyte apoptosis in DCM; inhibiting P53 may reduce DCM progression.
	Bcl-2	Mitochondrial outer membrane, MAMs	Inhibits apoptosis by regulating mitochondrial membrane permeability.	Increased Bcl-2 levels in DCM may serve as a compensatory anti-apoptotic mechanism.
	Bax	Mitochondrial outer membrane, MAMs	Promotes mitochondrial permeability transition pore (MPTP) opening and cytochrome c release.	Bax upregulation is associated with cardiomyocyte apoptosis in DCM; inhibiting Bax may slow DCM progression.
	OPA1	Mitochondrial inner membrane, MAMs	Affects cytochrome c release by regulating crista junctions (CJs).	Abnormal OPA1 processing causes excessive mitochondrial fission and DCM; regulating key proteases can improve cardiomyocyte function.
	Akt	MAMs, Cytoplasm	Phosphorylates IP3R3, lowering cell sensitivity to calcium-dependent apoptosis.	Reduced Akt activity accelerates cardiomyocyte apoptosis; restoring Akt activity may slow DCM progression.
	MTORC2	MAMs	Regulates Akt activity and influences apoptosis.	Loss of MTORC2 function reduces Akt activity, accelerating cardiomyocyte apoptosis; restoring MTORC2 function may slow DCM progression.
**Autophagy**	Parkin	MAMs, Cytoplasm	Regulates MFN2 ubiquitination and promotes a	Parkin deficiency impedes autophagy, leading to mitochondrial damage and DCM; restoring Parkin fun

			utophagosome formation.	ction may improve cardiomyocyte function.
	BECN1	MAMs, Cytoplasm	Promotes autophagy upon dissociation from Bcl-2.	BECN1 binds to Bcl-2 to suppress autophagy in DCM; restoring BECN1 function may slow DCM progression.
	AMPK	MAMs, Cytoplasm	Phosphorylates MFN2 to regulate MAMs' dynamic stability and activate autophagy.	AMPK activation promotes autophagy and protects cardiomyocytes; inhibiting AMPK may accelerate DCM progression.
**Necroptosis**	Nix	ER membrane, MAMs	Increases ER calcium storage and induces MPTP opening to trigger necroptosis.	Nix-mediated necroptosis is associated with DCM; inhibiting Nix may reduce DCM progression.
**Ferroptosis**	HMOX1	MAMs；ER membrane	Regulates iron homeostasis through heme degradation, releasing free iron (Fe^{2+}).	Overactivation causes mitochondrial iron overload, ROS, and ferroptosis, worsening myocardial injury. HMOX1 inhibition reduces damage, underscoring its therapeutic potential for DCM.
	FUNDC2	Mitochondrial outer membrane, MAMs	Regulates mitochondrial glutathione transporter SLC25A11 and GPX4 stability.	FUNDC2 regulates iron death by affecting mitochondrial GSH levels and is associated with DOX-induced cardiomyopathy.
